# The treatment of early breast cancer in women over the age of 70

**DOI:** 10.1038/bjc.2011.234

**Published:** 2011-06-21

**Authors:** A Ring, M Reed, R Leonard, I Kunkler, H Muss, H Wildiers, L Fallowfield, A Jones, R Coleman

**Affiliations:** 1 Brighton and Sussex Medical School, Sussex Cancer Centre, Royals Sussex County Hospital, Brighton BN2 5BE, UK; 2 Academic Surgical Oncology Unit, School of Medicine, University of Sheffield, Beech Hill Road, Sheffield, UK; 3 Cancer Services, Imperial College Healthcare NHS Trust, 3rd Floor North, Charing Cross Hospital, Fulham Palace Road, London W6 8RF, UK; 4 Edinburgh Cancer Centre, University of Edinburgh, Western General Hospital, Crewe Road, Edinburgh, Scotland, UK; 5 Lineberger Comprehensive Cancer Center, 170 Manning Drive, Campus Box 7305, Chapel Hill, NC 27599, USA; 6 University of Leuven, UZ Leuven, Campus Gasthuisberg, Herestraat 49, Leuven B–3000, Belgium; 7 Cancer Research UK Psychosocial Oncology Group, Brighton & Sussex Medical School, Brighton, UK; 8 Department of Academic Oncology, Royal Free Hospital, Pond Street, London NW3 2QG, UK; 9 Academic Unit of Clinical Oncology, University of Sheffield, Sheffield S10 2RX, UK

**Keywords:** breast cancer, elderly, geriatric oncology

## Abstract

One third of all breast cancers are diagnosed in women aged 70 or over. Older women are a heterogeneous population who are under-represented in clinical trials, and as a result uncertainty can exist as to what represents optimal treatment. This minireview, from an international authorship, summarises the existing evidence surrounding the management of early breast cancer in women aged 70 and over. The use of primary surgery and endocrine therapy, and adjuvant chemotherapy, radiotherapy, endocrine therapy and trastuzumab are discussed. Reference is made to ongoing clinical trials in this area and areas of controversy are highlighted.

In the United Kingdom one third of all breast cancers are diagnosed in women aged 70 or over, and with the ageing of the population the number of women in this age group will increase significantly over the next decade (Cancer Research UK, 2010). It is well documented that older women with breast cancer are less likely to be offered or receive standard treatment and that such undertreatment may impact on disease-specific mortality (Bouchardy *et al*, 2007; Lavelle *et al*, 2007). Older women are also a very heterogeneous group and are under-represented in clinical trials (Lewis *et al*, 2003). These factors lead to uncertainty as to what represents optimal therapy for these patients. This review summarises the current evidence base for the treatment of breast cancer in women aged 70 or over, highlighting where controversies exist.

## Surgery

Surgery is the mainstay of treatment in early breast cancer, but in older patients clinicians may have concerns about higher rates of anaesthetic and/or surgical complications. However, the vast majority of older patients are able to tolerate surgery and anaesthesia with very low morbidity and almost non-existent mortality (Wyld and Reed, 2007). When older women do undergo surgery, they are more likely to undergo mastectomy than younger women (Mustacchi *et al*, 2007) and are less likely to be offered or undergo breast reconstruction (NHS Information Centre, 2010). These observations may reflect patient choice or concerns over prolonged anaesthesia. However, it should be recognised that although cosmesis may be less important to some older women, most would still choose breast conservation surgery over mastectomy despite the need for radiotherapy (Sandison *et al*, 1996).

Axillary staging by axillary lymph node dissection has long been a standard of care in the surgical management of breast cancer. In many centres this procedure has been superceded by sentinel node biopsy, with surgical clearance only performed in those with positive nodes (although recent data have questioned the need for completion axillary dissection). Although older patients do not appear to be at increased risk of complications following axillary clearance, retrospective analyses and one randomised trial have questioned its value in older women with breast cancer (IBCSG, 1996). In a randomised trial, 473 women aged 60 or over with a clinically negative axilla were randomly assigned to undergo primary surgery plus axillary clearance followed by tamoxifen, or surgery without axillary dissection followed by tamoxifen (IBCSG, 1996). At a median follow-up of 6.6 years there were no differences between the two groups in disease-free or overall survival. Interestingly in the ALMANAC trial (comparing sentinel node biopsy and standard axillary treatment), which used validated measures of patient reported quality of life and arm morbidity, older women (65 years and over) irrespective of axillary management had better quality of life outcomes at all stages of the 18-month follow-up than younger women (Fleissig *et al*, 2006).

However, perhaps a more fundamental question is whether surgery can be omitted and replaced by primary endocrine therapy. In a Cochrane review examining studies comparing surgery with or without adjuvant tamoxifen and primary endocrine therapy with tamoxifen alone, there was no significant difference in overall survival, but tamoxifen alone was inferior to surgery in terms of local disease control (Hind *et al*, 2007). Studies in this review recruited women of 70 years or over who were fit and well, and in some studies patients were not selected for ER status. The average period of cancer control before progression was 18 to 24 months. Therefore the survival of very old and frail patients with confirmed ER-positive disease is unlikely to be adversely affected by the decision to avoid primary surgery. Subsequently, the question has arisen as to whether aromatase inhibitors, which have proven slightly superior to tamoxifen in both the metastatic and adjuvant settings, may be a more appropriate alternative to surgery. It was planned to address this prospectively in the ESTEEM (endocrine ± surgical therapy for elderly women with mammary cancer) trial. ESTEEM was a large United Kingdom trial that aimed to recruit 1200 patients aged 75 years or over with primary operable oestrogen-receptor (ER)-positive breast cancer, and randomise them to receive primary endocrine therapy with anastrozole or surgery plus adjuvant anastrozole. A key component of this trial was the inclusion of frail patients who could undergo surgery under local anaesthetic and the extensive use of geriatric assessments, including measurements of co-morbidities, cognitive function and functional status, in order to characterise better those who might benefit. Unfortunately, ESTEEM closed early owing to slow recruitment. The early closure of ESTEEM and other studies in this population of patients (see below) demonstrates the challenges associated with non-blinded studies, with major differences between the groups and a direct comparison of the approaches is not likely to be forthcoming.

At present, the optimal approach is to use standard surgical approaches including SNB for all patients with estimated survivals of at least several years. However, primary endocrine therapy is a reasonable alternative for patients with a limited life expectancy (<2–3 years) due to multiple co-morbidities or extreme old age.

## Adjuvant radiotherapy

Adjuvant radiotherapy is the current standard of care for patients with early breast cancer following breast-conserving surgery, and in patients with a high risk of local recurrence following mastectomy. According to the Oxford overview analysis: in women undergoing breast-conserving surgery, 5-year local recurrence risks were 7 *vs* 26% (2*P*<0.00001) and a 15-year breast cancer mortality risks 30.5 *vs* 35.9% (2*P*=0.0002), in those who underwent radiotherapy following surgery compared with those undergoing surgery alone (EBCTCG, 2005a). Radiotherapy following breast-conserving surgery was associated with similar proportional reductions in local recurrence across all age groups. However, the absolute benefits of treatment were lower in older patients as their overall risk of local recurrence was less (absolute reductions in 5-year local recurrence risk for post-BCS radiotherapy: 22, 16, 12 and 11% for those aged <50, 50–59, 60–69 and ⩾70 years, respectively; test for trend in absolute benefits 2*P*=0·00002). There was, however, also an excess of non-breast cancer mortality in women undergoing radiotherapy, largely from heart disease (rate ratio 1.27, 2*P*=0.0001) and lung cancer (rate ratio 1.78, 2*P*=0.0004). It seems likely that these risks are lower with more modern radiotherapy techniques, although older patients probably remain those at greatest risk.

One important large randomised trial has directly addressed the role of adjuvant radiotherapy following breast-conserving surgery in older women (Hughes *et al*, 2010). In this study women aged 70 years or more with T1 N0 hormone receptor-positive breast cancers who had undergone breast-conserving surgery and were being treated with tamoxifen, were randomised to receive radiotherapy or not. At the most recent update (at a median follow up of 10.5 years), the rate of local or regional recurrence was 9% in those treated with tamoxifen alone, and 2% in those who also received radiotherapy (*P*<0.001). There were no significant differences between the two groups in overall survival.

Aromatase inhibitors have a favourable impact on local recurrence when compared with tamoxifen (ATAC Trialists, 2008). Taken together with recent trends for falling local recurrence rates, these factors are likely to further reduce the already low local recurrence rates seen in older patients, and may mean that the absolute benefits of radiotherapy in this population in the future will be smaller still. The PRIME II (post-operative radiotherapy in minimum-risk elderly phase II) trial has recruited women aged 65 or over with low-risk breast cancer (⩽3 cm, grade I or II, node negative and hormone-receptor positive), treated by breast-conserving surgery and adjuvant endocrine therapy (Kunkler, 2004). Patients were randomised to receive post-operative breast radiotherapy or no radiotherapy, and the trial was designed to detect a difference in local recurrence rates of at least 5%. The trial completed recruitment in November 2009 and the results were awaited.

At present post-operative radiotherapy remains the standard of care in older patients undergoing breast-conserving surgery, except in those very frail patients with a limited life expectancy. It is frequently reported that older patients are much more likely to be treated with breast-conserving surgery alone (Lavelle *et al*, 2007, Schonberg *et al*, 2010). The reasons are likely to be multifactorial, including issues relating to the surgeon and the oncologist, service practicalities and patients’ wishes. However, older patients with tumours at higher risk for local recurrence and reasonable life-spans should be offered irradiation.

Over recent years two studies have compared a previous standard schedule of 50 Gy in 25 fractions with shorter schedules of 15–16 fractions over 21 and 22 days, respectively (Whelan *et al*, 2010, START Trialists, 2008). In these studies 11.5 and 17% of patients were aged 70 or over. There were no significant differences in efficacy between the treatment strategies, with the shorter fractionation schedule providing convenience to patients and reduced healthcare expenditure. In the future, hypofractionation, partial breast irradiation, brachytherapy and intraoperative radiotherapy, may all mean that radiotherapy can be more efficiently delivered. Such developments involving fewer visits may be of particular relevance to older patients.

## Adjuvant chemotherapy

Compared with their younger counterparts, older women are more likely to have low-grade, oestrogen receptor-positive tumours (Mustacchi *et al*, 2007). As a consequence, endocrine therapy has been the mainstay of adjuvant systemic therapy for the older age group. However, a significant proportion of older women do still present with tumours with adverse prognostic features, suggesting a significant risk of disease recurrence (Mustacchi *et al*, 2007). The current life expectancy of a 70-year-old woman in the United Kingdom is 16 years. Therefore many older women with breast cancer are at risk of recurrence and death from breast cancer in their projected lifetime, and may potentially benefit from chemotherapy (Office for National Statistics, 2010).

In the Oxford overview of the Early Breast Cancer Trialists’ Collaborative Group, published in 2005, the benefits of polychemotherapy were seen to decrease progressively with increasing age. The reductions in risk of death for polychemotherapy compared with no chemotherapy were 30, 15, 9 and 13% for the age groups 40–49, 50–59, 60–69 and 70 and over, respectively (EBCTCG, 2005b). Hence the benefit for patients aged 70 or over was of the same order as for younger postmenopausal women, but this result was not significant as the number of older women in the analysis was small (only 4% of women included in the trials analysed were aged 70 years or over) (EBCTCG, 2005b). The value of programmes such as Adjuvant! Online may be limited in this patient population, as the predictive estimates are based on clinical trials where older patients are under-represented. The role of gene expression analyses such as Oncotype Dx (Genomic Health, Redwood City, CA, USA) and Mammaprint (Agendia BV, Amsterdam, The Netherlands) to help identify those most likely to benefit from adjuvant chemotherapy may be of particular help in older patients, but to date no studies have been conducted specifically in this age group.

Two contemporary trials, adjuvant cytotoxic chemotherapy in older women (ACTION) and chemotherapy adjuvant study for women at advanced age (CASA), planned to examine the benefits of adjuvant chemotherapy in women aged over 70 years and 65 years, respectively, with ER-negative or high-risk ER-positive tumours. Both trials included chemotherapy and no chemotherapy arms, in an attempt to quantify the absolute gains from chemotherapy. Unfortunately both trials closed due to poor recruitment. Therefore, the available data suggest that there may be a benefit to adjuvant systemic chemotherapy in some older women with breast cancer, but which patients benefit and the magnitude of the benefit are unknown. Table 1 summarises current and recently closed trials of adjuvant chemotherapy in older women.

The Cancer and Leukemia Group B (CALGB) 49907 study took as a premise that adjuvant chemotherapy is of benefit in older women, and has compared two treatment regimens (Muss *et al*, 2009). Women aged 65 or older with invasive T1–T4 breast cancer (ER positive or negative) and node positive or negative, were randomised to receive standard chemotherapy (six cycles of CMF or four cycles of AC, according to physician choice), or six cycles of oral capecitabine. Accrual stopped at 633 patients, at which point 61% of patients were aged 70 years or over. After a median follow-up of 2 years, patients randomised to capecitabine were 2.4 times more likely to experience a relapse-free survival event (95% CI: 1.5–3.8; adjusted *P*=0.0003) and 2.1 times more likely to die (95% CI: 1.2–3.7; *P*=0.02) than those receiving standard chemotherapy. This trial shows that there is a benefit to giving patients over 65 with a good performance status standard polychemotherapy compared with single agent capecitabine chemotherapy. An unplanned subset analysis in this trial showed that the major benefit for standard chemotherapy was in patients with hormone receptor-negative tumours.

In younger patients dose-dense adjuvant chemotherapy regimens are often used, however, in the pivotal CALGB 9741 trial demonstrating the benefits of the dose-dense approach only 3% of patients were aged 70 or over (Citron *et al*, 2003). The concurrent dose-dense regimens were associated with more red-cell transfusions, more grade-3 emesis and more post-chemotherapy neurotoxicity than the sequential approach, all of which may be particularly relevant in older patients. Toxicity is likely to be a key factor when considering adjuvant chemotherapy in older patients. In a large retrospective review of four CALGB trials of adjuvant chemotherapy, treatment-related mortality was found to be related to age: 0.2% (⩽50 years), 0.7% (51–64 years) and 1.5% (⩾65 years) (*P*<0.001) (Muss *et al*, 2005). Age is also a significant risk factor for congestive cardiac failure in women receiving adjuvant chemotherapy for breast cancer (HR, 1.79 per 10 years; 95% CI: 1.66–1.93) (Pinder *et al*, 2007). The US Oncology group has compared an anthracycline (doxorubicin/cyclophosphamide) with a non-anthracycline (docetaxel/cyclophosphamide) regimen in the adjuvant treatment of operable breast cancer (Jones *et al*, 2007). The non-anthracycline arm was found to be superior to the anthracycline treatment, including in the 16% of patients who were aged 65 or over at study entry. Owing to the low risks of cardiotoxicity and acceptable neutropaenic sepsis rate, this regimen has become popular with many oncologists treating older women. It should be emphasised, however, that much of the available data regarding treatment-related toxicities relate to clinical trial populations of older women with breast cancer. It is likely that in the less-selected general population of older women with breast cancer that toxicity rates may be even higher.

## Endocrine therapy

The efficacy of adjuvant endocrine therapy in older patients is well established. In women with ER-positive breast cancer, 5 years of adjuvant tamoxifen reduces the annual risks of recurrence and breast cancer mortality by 39 and 31%, respectively, irrespective of age (<50, 50–69, ⩾70 years (EBCTCG, 2005b). With the emergence of aromatase inhibitors in the adjuvant setting in postmenopausal women, clinical trials of endocrine therapy have inevitably studied older populations. In the ATAC and BIG 1.98 studies, the benefits of aromatase inhibitors over tamoxifen in the adjuvant treatment of breast cancer were seen across all age groups (ATAC Trialists, 2008 and Crivellari *et al*, 2008). However, in the BIG 1-98 study older patients (⩾75 years) were less likely to complete trial treatment, although the rates of discontinuation were similar in the letrozole and tamoxifen treatment groups (Crivellari *et al*, 2008). The well-described increased bone loss during treatment with aromatase inhibitors coupled with the typical decline in bone mineral density (BMD) with age, makes attention to bone health of particular importance in the older patient. Bone protection with a bisphosphonate is required not only in those with low BMD (T score <−2), but also in patients over 75 with one or more additional risk factors for fracture (Reid *et al*, 2008). Bisphosphonates have been shown to prevent bone loss during treatment with aromatase inhibitors, including in those with pre-existing osteoporosis (van Poznak *et al*, 2010). Therefore decisions on choice of endocrine therapy in the elderly, as in younger patients, should be based on the biology and risk of recurrence of the underlying breast cancer, rather than the level of BMD. Other than bone health other differences in the relative toxicity profiles of aromatase inhibitors, such as with regard cerebrovascular, thromboembolic events and potentially musculoskeletal complaints may influence choice of agent.

## Adjuvant trastuzumab

The proportion of breast cancers which are HER2 positive appears to be similar or slightly lower than that seen in younger women with around 10–16% of tumours in women aged 65 and over testing positive (Mustacchi *et al*, 2007). Only 16% of the patients entering the HERA and NSABP B31/N9831 trials were aged 60 or over, but the benefits of trastuzumab in this subgroup appeared to be same as for the whole-study populations (Smith *et al*, 2007 and Romond *et al*, 2005). Trastuzumab-induced cardiac dysfunction might be a particular concern in older patients. In the HERA trial the incidence of cardiac adverse events for those 60 years or over, compared with those less than 60 years was not significantly different (3.75 *vs* 3.61% difference in incidence 0.14% (95% CI: −2.54-2.80)) (Suter *et al*, 2007). Moreover, trastuzumab-induced cardiac toxcity is usually reversible. However, the number of patients over the age of 60 was small, and patients with known cardiac disease or a left ventricular ejection fraction of less than 55% after chemotherapy were excluded. Therefore although it would be reasonable to conclude that age is not a risk factor for cardiac dysfunction, there are limited data and a significant number of elderly patients may not be eligible for treatment with trastuzumab based on pre-existing co-morbidities. Measures to reduce the risks of cardiac toxicity such as the use of non-anthracycline regimens such as the docetaxel, carboplatin and trastuzumab (‘TCH’) regimen, and ongoing trials examining shorter durations of adjuvant trastuzumab may be particularly relevant in older patients. Although some clinicians do use trastuzumab without chemotherapy in the adjuvant setting, there are no data to support this approach.

## Conclusions

The data discussed in this review and the information emerging from ongoing clinical trials provide a framework for the management of breast cancer in older women. In essence, older patients should be treated according to tumour biology as for younger patients, except where co-morbidities or frailty are likely to mitigate the benefits of treatment or mean that treatment will not be tolerated. However, there are some fundamental questions regarding everyday management, which remain unanswered (Table 2). Perhaps, the biggest challenge is to apply the available evidence in the clinic to the individual older patient. Patients over the age of 70 represent a highly heterogeneous population in terms of co-morbidities, fitness, life expectancy, social situation, cognitive function and desire for treatment. Assessment of these factors taking into account the risks of death from competing causes of mortality and the likely tolerance of any proposed treatment is a vital component to making appropriate informed decisions regarding adjuvant treatment. Assessment tools to do this have been developed, but most require validation in patients with cancer (Extermann and Hurria, 2007). It is imperative that we do this in order that we can best serve the ageing population of women with breast cancer.

## Figures and Tables

**Table 1:** Randomised trials of adjuvant chemotherapy in older women with breast cancer

**Name/group**	**Study population**	**Control arm**	**Experimental arm**	**Status**
ACTION (UK NCRN)	Age ⩾70. ER negative or high-risk ER positive.	If ER negative: nil. If ER positive: endocrine therapy.	Four cycles AC or EC. 2nd randomisation 2 weekly *vs* 3 weekly with GCSF.	Closed owing to poor recruitment.
CALGB 49907	Age ⩾65. LN positive or high-risk LN negative. Any ER.	Four cycles AC or six cycles CMF. If ER positive: endocrine therapy.	Six cycles capecitabine. If ER positive: endocrine therapy	Completed and published (Muss *et al*, 2009).
CASA (IBCSG/BIG)	Age ⩾65. LN positive or negative. ER negative.	Nil or low-dose metronomic methotrexate.	Eight cycles of pegylated liposomal doxorubicin (2 weekly)	Closed owing to poor recruitment.
ELDA (National Cancer Institute, Naples)	Age 65–80. ER and PgR negative or LN positive, or >20 mm, or G2/3.	IV CMF (four or six cycles)	Weekly docetaxel (days 1, 8, 15 q 28) (four or six cycles)	Open to recruitment.
ICE (German Breast Group)	Age⩾65. LN positive or high-risk node negative. Any ER status.	Ibandronate. If ER positive: anastrazole.	Ibandronate and six cycles capecitabine. If ER positive: anastrozole.	Closed to recruitment: efficacy results awaited.
ICE II (German Breast Group)	Age⩾65. pT1/2 and pN0/1 with an increased risk based on either uPA/PAI1 or clinico-pathological risk parameters.	Four EC or six CMF	Six cycles of weekly nab-paclitaxel (days 1, 8, 15 q22 with a week of rest every 6 weeks) with capecitabine (days 1–14, q21)	Open to recruitment.

Abbreviations: AC=doxorubicin/cyclophosphamide; ACTION=adjuvant cytotoxic chemotherapy in older women; CALGB=Cancer and Leukemia Group B; CASA=chemotherapy adjuvant study for women at advanced age; CMF=cyclophosphamide, methotrexate and 5-fluorouracil; EC=epirubicin/cyclophosphamide; ER=oestrogen receptor; LN=lymph node; PgR=progesterone receptor.

**Table 2:** Current controversies in the management of women aged 70 or over with breast cancer

*Surgery*
The role of screening tools and comprehensive geriatric assessment (CGA) in selecting patients for primary endocrine therapy compared with surgery.
The role of sentinel node biopsy in the staging of older women with breast cancer.
The need for further treatment in older patients with a positive sentinel node biopsy.
Patient information seeking and decision-making preference for surgical treatment including reconstruction and primary endocrine therapy.

*Adjuvant radiotherapy*
The role of adjuvant radiotherapy following wide local excision in older women with low risk of local recurrence.
Optimal fractionation schedule.
Role of boost to tumour bed.
Role of newer radiotherapy techniques, including intra-operative techniques.

*Adjuvant chemotherapy*
The role of screening tools and CGA in selecting patients for adjuvant chemotherapy.
The absolute benefits derived from adjuvant chemotherapy in older women and the role of gene expression assays in selecting therapy.
The most appropriate chemotherapy regimen for women aged 70 and over.
The toxicity of treatment in the non-trial population.
